# Books for Review

**Published:** 1891-01-31

**Authors:** 


### Books for Review.

Dr. Hyde's "Guide to Massage" recently noticed in The Hospital is published by John Heywood, Deansgate, Manchester.

